# Blood Transfusion Procedure: Assessment of Serbian Intensive Care Nurses’ Knowledge

**DOI:** 10.3390/healthcare12070720

**Published:** 2024-03-25

**Authors:** Dragana Simin, Vladimir Dolinaj, Branislava Brestovački Svitlica, Jasmina Grujić, Dragana Živković, Dragana Milutinović

**Affiliations:** 1 Department of Nursing, Faculty of Medicine, University of Novi Sad, 21000 Novi Sad, Serbia; vladimir.dolinaj@mf.uns.ac.rs (V.D.); branislava.brestovacki@mf.uns.ac.rs (B.B.S.); dragana.zivkovic@mf.uns.ac.rs (D.Ž.); dragana.milutinovic@mf.uns.ac.rs (D.M.); 2 Department of Anesthesia and Intensive Care, University Clinical Centre of Vojvodina, 21000 Novi Sad, Serbia; 3 Institute for Child and Youth Health Care of Vojvodina, 21000 Novi Sad, Serbia; 4 Department of Transfusiology, Faculty of Medicine, University of Novi Sad, 21000 Novi Sad, Serbia; jasmina.grujic@mf.uns.ac.rs; 5 Vojvodina Blood Transfusion Institute, 21000 Novi Sad, Serbia

**Keywords:** nurses, transfusion, knowledge, intensive care

## Abstract

Many patients require administering one or more blood components during hospitalisation in the Intensive Care Unit (ICU). Therefore, nurses’ knowledge of who is responsible for immediately administering blood transfusions, monitoring patients, and identifying and managing transfusion reactions is crucial. This cross-sectional descriptive-analytical study aimed to assess the knowledge of ICU nurses in tertiary healthcare institutions about blood transfusion procedures. The questionnaire about the transfusion procedure was designed and reviewed by experts. The questionnaire consisted of 29 items divided into three domains. The scores on the knowledge test ranged from 10 to 27. Generally, 57.7% of nurses had moderate, 23.4% low, and 18.9% high levels of knowledge about the transfusion procedure. Most nurses answered correctly about refreezing fresh frozen plasma, verifying the transfusion product, and identifying the patient. Of the nurses, 91.0% would recognise mild allergic reactions, and 98.2% knew about the supervision of sedated patients. Nurses showed poor knowledge of the length of usage of the same transfusion system for red blood cells, labelling, and transfusion administration in febrile patients. Nurses with higher education and longer working experience had significantly better outcomes (*p* = 0.000) on the knowledge test. Continuous education of ICU nurses on safe transfusion usage is recommended.

## 1. Introduction

Intensive care units (ICU) are units in which intensive and specialised medical and nursing care to critically ill patients is provided. In these units, an enhanced capacity for monitoring and multiple modalities of physiologic organ support to sustain life through life-threatening organ system insufficiency is enabled [1]. During hospitalisation in the ICU, many patients require the administration of one or more blood components due to ongoing blood loss or haemostatic disorders [2]. When deciding on red blood cell (RBC) transfusion, the following should be taken into account: cause and stage of anaemia, patient comorbidities and age, conditions where there is increased need for oxygen (sepsis), and blood loss [3]. Transfusion trigger is defined as the haemoglobin (Hb) value below which RBC transfusion is indicated. A restrictive transfusion strategy seeks to maintain a lower haemoglobin level (70–90 g/L) with a transfusion trigger when the haemoglobin drops below 70 g/L, whereas a liberal transfusion strategy aims to maintain higher haemoglobin (100–120 g/L), with a threshold for transfusion when haemoglobin drops below 100 g/L) [4]. Fresh frozen plasma (FFP) is indicated to substitute coagulation factors in individuals receiving massive transfusions, to reverse warfarin’s effect urgently, to treat known coagulation factor deficiency, and in cases of thrombotic thrombocyte thrombocytopenia purpura [5]. Platelet transfusion is usually required in a bleeding patient below a platelet count of 50 × 10^9^/L but rarely above 100 × 10^9^/L. If the values fall between these two, transfusion is considered in cases of platelet dysfunction, ongoing bleeding, and surgeries such as those in the eye and brain [5].

According to the literature, approximately 15–53% of ICU patients receive transfusions [6]. In the United States, in 2021, more than 1.7 million red blood cell units were transfused in ICUs [7]. However, data on the number of administered blood units and the incidence of transfusion in ICU patients in Serbia have not been published.

Blood and blood components perform therapeutic responses and, at the same time, are capable of causing significant adverse effects, which can lead to the deterioration of the critically ill patient’s health. The administration of blood products is commonly associated with transfusion reactions, which occur in up to 1 out of every 100 transfusions [8]. Reports show that the incidence of transfusion reactions of RBCs and FFP is 1.7–4.3 per 100,000 transfusions and 62.6 per 100,000 in platelets [9]. The severity of adverse events varies from mild, which could include generalised discomfort, fever, tachycardia, rash, and hypotension, to severe, which could result in anaphylactic reactions and acute haemolytic reaction (AHTR); these could threaten the patient’s life [10]. Therefore, transfusion of blood and blood products in the ICU should be considered as administration of any other medication. Thus, before using the blood and blood components in critically ill patients, their use’s benefits and risks must be considered [11].

The administration of blood and blood components in the ICU is a multidisciplinary procedure. The multidisciplinary team consists of an anaesthesiologist–intensivist, transfusiologist, and nurse. In the ICU, nurses are responsible for immediate blood transfusions, monitoring patients, and identifying and managing transfusion reactions [12,13].

There are several levels of education for nurses in Serbia. Four-year secondary medical schools provide the first level of nursing education, and higher degrees (bachelor’s, master’s and doctoral) are obtained at university [14]. Theoretical and practical aspects of the blood transfusion procedure are studied at all levels of education, but to varying degrees. However, regardless of the level of professional training for independent work in the health system, nurses in Serbia must have a license issued by the Chamber of Nurses. The license duration is limited to seven years, and the extension is conditioned by regular attendance of continuing education courses.

Due to the different levels of education of nurses employed in our institution, assessing nurses’ knowledge regarding the transfusion of blood and blood products in critically ill patients is of extraordinary importance in ensuring the safety and effectiveness of the transfusion procedures. So far, we know that no similar studies have been carried out in the Republic of Serbia or the southeast region of Europe. Concerning all the issues mentioned above, the study aimed to assess the knowledge of ICU nurses about blood transfusion procedures. In addition, we wanted to identify predictors that might influence their knowledge.

## 2. Materials and Methods

### 2.1. Study Design and Settings

A cross-sectional descriptive-analytical study was conducted at the Intensive Care Unit (ICU) of the Clinic of Anesthesia, Intensive Care and Pain Therapy of the University Clinical Center of Vojvodina, in October and November 2022. The University Clinical Center of Vojvodina is one of the regional tertiary healthcare institutions. It provides healthcare services for the area of Vojvodina (a northern province in Serbia) and has 39 beds for intensive care to patients who are critically ill or injured.

The study adhered to Strengthening the Reporting of Observational Studies in Epidemiology (STROBE) guidelines.

### 2.2. Sample

A purposive sample consisted of Intensive Care Unit nurses. Using sample size software for cross-sectional studies (www.calculator.net; accessed 1 October 2022), it was determined that a sample of 111 nurses was required for a 95% confidence interval, with a margin of error of 0.05.

**Figure 1:**
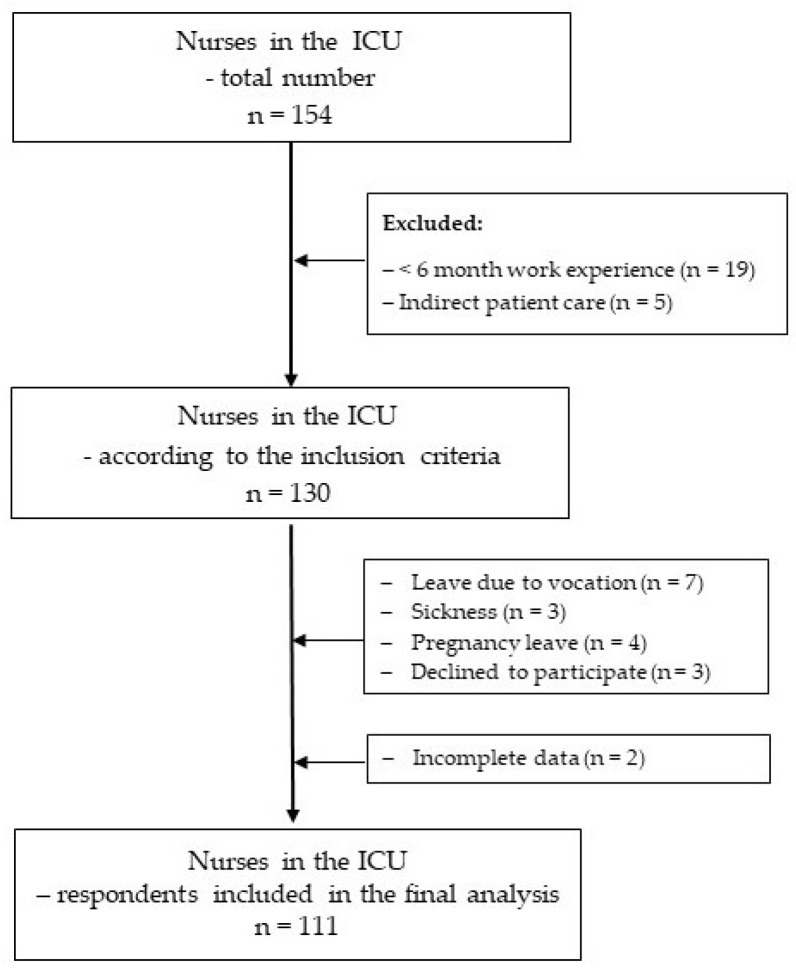
Flowchart summary of data collected from nursing study participants.

The criterion for inclusion in the study was that nurses were privileged to direct patient care, had a minimum of 6 months of work experience in the ICU (Figure 1), and voluntarily signed written consent to participate in the study.

### 2.3. Instrument

The questionnaire on nurses’ knowledge of the blood transfusion procedure and a general questionnaire containing data about gender, age, education, and work experience were used as the study instruments.

The researchers designed the questionnaire on nurses’ knowledge of the blood transfusion procedure based on the World Health Organization (WHO) Clinical Transfusion Procedure [15], National Guidelines for Clinical Use of Blood [16], and earlier study [17,18,19,20]. Before use, the questionnaire was reviewed by a panel of experts consisting of three nurses with academic education, more than ten years of work experience in the ICU, two doctors, one specialist in anaesthesia and intensive care, and one specialist in transfusionology. According to their assessment, the content of the questionnaire was appropriate. The questionnaire consisted of 29 items divided into three domains about nurses’ knowledge related to pretransfusion steps (items 1–9) at the beginning of the application of transfusion (items 10–17), and application of transfusion, transfusion reactions (18–29). Nine items (2, 5, 6, 8, 14, 16, 19, 25, and 26) had a true–false answer option, while others were multiple-choice options. Each correct answer is scored with one point. The maximum total score was 29, 9 for the first domain, 8 for the second, and 12 for the third domain of the questionnaire. A score of less than 15 was considered low, 15 to 22 moderate, and a score equal to or greater than 23 indicated a high nurse knowledge level about blood transfusion. The internal consistency was confirmed using the Cronbach’s alpha coefficient (α = 0.72) and the Spearman–Brown coefficient (r = 0.79).

### 2.4. Data Collection

At the end of the day shift, the researchers distributed paper questionnaires with an information letter and an informed consent form. The nurses who agreed to participate in the study had to sign the informed consent form, and after completing the anonymous questionnaire, nurses had to insert it through the slot into the locked box. In each ICU, nurses are provided an area to fill in the questionnaire independently. They were asked to put down their mobile phones, and the time to complete the questionnaire was not limited.

### 2.5. Data Analysis

Using IBM statistical software, version 26.0, statistical data processing was performed only for questionnaires where answers were given to all items. Depending on the nature of the variable, descriptive statistics methods were used to determine absolute frequency with appropriate percentages, mean values (M), and standard deviation (SD). The difference between the two groups was compared with the t-test, the Mann–Whitney test, while the one-factor analysis of variance (ANOVA) was used to compare the mean values of several groups. Effect sizes (d and η^2^) were calculated to quantify the obtained differences. Standard multiple linear regression analysis was used to predict the factors influencing nurses’ knowledge. For all analyses, *p* < 0.05 was considered statistically significant.

### 2.6. Ethical Consideration

The implementation of this study was approved by the Ethics Committee of the University Clinical Center of Vojvodina (Ref. No. 00-1206/2021). The nurses’ consent to participate in the study was obtained following the Declaration of Helsinki

## 3. Results

### 3.1. Sociodemographic Characteristics of the Study Sample

Most nurses, 93 (83.8%), were female, and 86 (77.5%) had a secondary medical school diploma. The sociodemographic characteristics of nurses for the whole sample and the level of professional education are shown in Table 1.

### 3.2. Analysis of Nurses’ Knowledge of the Blood Transfusion Procedure

Table 2 shows the distribution of correct answers for each item in the questionnaire.

Analysis of individual items from the pretransfusion steps domain showed that most correct answers were for the item about refreezing fresh frozen plasma (81.1%). The least correct answers were given to the item about the time and place of filling in the label (23.4%).

Almost all nurses knew how to accurately verify the correctness of the transfusion product and determine the patient’s identity at the beginning of the transfusion (99.1%). This item from the domain of initiation of transfusion is also the item with the highest number of correct answers in the entire questionnaire. Also, more than 50% of nurses correctly answered seven out of eight items in this domain. Meanwhile, 45% of nurses were aware that the same transfusion system could be used for 2 to 4 units of red blood cells (RBCs).

The results related to transfusion administration and transfusion reaction show that the least correct answers in this domain, and the entire questionnaire, were to the item about the administration of transfusion in febrile patients in the ICU. In contrast, 91.0% of nurses correctly marked the first signs of a mild allergic reaction, and 98.2% were sure of the supervision required by a sedated patient during a transfusion.

### 3.3. Total Score on the Knowledge Test

The scores on the knowledge test ranged from 10 to 27 (mean 17.70, SD 4.28). The mean values for each knowledge domain are shown in Table 3.

More than half of the nurses (57.7%) had a moderate level of knowledge, 26 (23.4%) had a low level of knowledge, and 21 nurses had a high level of knowledge about the transfusion procedure. The data in Table 4 indicate that a low level of knowledge was not recorded among nurses with more than five years of work experience and a BA.

### 3.4. Univariate Analysis of Mean Values on the Knowledge Test to the Sociodemographic Characteristics of Nurses

Table 5 shows the differences in mean values on the knowledge test for the entire questionnaire and according to domains. The results of the Mann–Whitney U test showed that the observed differences in all domains of transfusion knowledge to gender were not significant.

On the contrary, the t-test confirmed that the observed higher values of the score on the knowledge test achieved by nurses with higher education differ significantly from those with secondary education. The differences in the scores for the entire questionnaire and in all domains of knowledge related to this sociodemographic characteristic were very significant (*p* = 0.00). Also, Cohen’s d values indicate a large effect of education on obtained scores.

Nurses with five or more years of work experience had the best knowledge on the test. The significance of this difference was not confirmed by a one-factor analysis of variance for the pretransfusion steps domain (*p* = 0.425). At the same time, the observed differences for the total score and other knowledge domains were very significant (*p* = 0.00); according to Cohen’s d values, they had a large effect.

### 3.5. Standard Multiple Regression Analysis of the Effect of Sociodemographic Characteristics on the Knowledge Test Score

The results obtained by standard multiple regression analysis indicate that our model, which included the level of education and work experience, explains 60.6% of the variance in the total score on the transfusion knowledge test (Table 6). Although both variables make a statistically significant contribution, the highest contribution is made by the level of education (Beta = 0.710).

## 4. Discussion

Blood transfusion is a common procedure in the ICU. Many factors contribute to ICU patients being frequent recipients of allogeneic blood transfusions [11]. During their ICU stay, 15% to 53% of patients receive a transfusion [6]. The results of a recent international prospective study conducted in 30 countries where data from 233 ICUs were analysed showed that 25% of patients received one or more units of red blood cells (RBCs) [7]. Proper use of blood transfusion and blood derivatives saves lives and improves the health of many people [15]. Like any therapeutic procedure and transfusion, there are several potential risks to patient safety. The basis for minimising these risks is that the transfusion is applied by trained and experienced personnel and by rigorous adherence to the entire procedure process [21].

More than half of the nurses in this study had a moderate level of knowledge about blood transfusion. Earlier studies also showed that nurses’ knowledge of this procedure was low or moderate [12,13,17,18,20,22,23,24,25,26]. Since transfusion of blood and blood products is a multistage procedure, our studies and those mentioned above aim to be used to identify risky parts of the transfusion process and at-risk populations of blood recipients to ensure safe blood administration in intensive care units.

One of the first steps in safely and properly administering transfusion involves providing the right blood to the right patient at the right time [15,16]. The results of our study showed that almost a third of nurses have adequate knowledge regarding medical orders and patient identification verification. However, only 26 (23.4%) of our nurses marked all the data that the label must contain and that the data on the label should be filled in at the patient’s bedside immediately after taking the blood sample. This step in the transfusion procedure is extremely important because of the risk that the patient’s blood sample will end up in the wrong sample test tube. The sample test tube should not be labelled before taking the sample [15]. When the blood in the sample test tube is not the blood of the patient whose information is on the label, it can lead to fatal outcomes due to the transfusion of ABO-incompatible blood [27].

The application of information technology can significantly contribute to reducing this and other errors related to blood transfusion [12]. Currently, the barcoding system is commonly used but is slowly being replaced by a radio-frequency identification tag system. Unfortunately, the price often limits their application [27]. The financial aspects of the institution where the study was conducted significantly prevent the implementation of many modern technologies in the entire clinical care process. The application of barcode technology started a few years ago in certain areas of laboratory diagnostics. However, this system has not yet covered all segments of patient care during hospitalisation. The impact of the human factor can be reduced by applying modern technologies, but they alone cannot completely remove all risks [6,27]. At the same time, a project implemented in the Basque Country showed that regional information systems that generate all relevant information in all stages of the blood transfusion process can significantly increase patient safety and transfusion efficiency [28]. Considering the results obtained with this research of our ICU nurses and our institution’s technical (in)ability in order to reduce the risk of fatal errors in pretransfusion time, multiple interventions should be undertaken, such as education of nursing staff, raising awareness about the causes and impact of human error on patient safety, and the rigorous control of the superior nurses [27].

The nurses in our study had a high level of knowledge about transporting blood from the blood bank to the ICU (79.3%). However, slightly more than half of the nurses (58.6%) stated that constant agitation is necessary when transporting concentrated platelets longer than 2 h. Such answers can perhaps be explained by the fact that the service for the blood bank and the ICU is not far away and that the transfer does not take more than 15 min. At the same time, it should not be overlooked that only a third of the nurses marked concerted platelets as the derivative with the highest risk for infection. Namely, to preserve the function of platelets, they are not cooled but stored at a temperature of 22 °C to 24 °C, which increases the risk of proliferation of bacteria and other microorganisms [15,16].

The obtained results suggest that future activities should increase patient safety during blood transfusion and focus on nursing interventions regarding healthcare-acquired infection (HAI). Moreover, this aspect requires special attention because the risk of HAIs is twice as high in ICU patients compared to patients from other clinical departments [29]. The high prevalence of invasive procedures and therapeutic modalities, the use of immunosuppressive drugs, and the presence of comorbidities significantly contribute to the fact that patients in the ICU have this level of risk of HAI [30]. According to Edwardson [29], blood transfusion is one of the factors that can increase the risk of HAIs in the ICU. Nurses can significantly contribute to the implementation of HAI prevention measures in this, as well as in other domains of patient care in the ICU. Namely, of all multidisciplinary team members in the ICU, nurses are more often in direct contact with the patient [30].

Analysis of responses of our ICU nurses about how to thaw FFP and refreeze once-thawed FFP showed that the nurses in this study had a high level of knowledge. However, our nurses, as well as nurses in several other studies, did not have enough knowledge about storing other blood products and returning unused products to the blood bank [18,19,20,31,32,33]. Freixo et al. [31], in a critical analysis of the nurses’ responses, state that this can negatively affect the quality of blood products and the adequate management of supplies. Namely, two crucial rules for the storage of blood products are the 30 min rule, the period in which the product must be used since it is not stored in a temperature-controlled environment, and the 4 h rule, the time within which the transfusion should be completed [15].

The nurses in our study knew the principles of good practices related to verifying the blood product’s correctness and the patient’s identity immediately before the start of the transfusion. Namely, almost all nurses (99.1%) correctly marked all necessary elements of verification and identification. The same results were obtained in the studies of Uzun et al. [26]. The literature data indicate that these procedures may be absent in patients with active bleeding and when nurses start the transfusion after the shift [23,24]. Also, the reason for the incorrect answer regarding blood product verification was the practice in which only one nurse performed the check [19]. These findings indicate that potentially skipping certain procedures would increase the risk for the safe application of transfusion; that is, the chance that the right blood will not be given to the right patient is increased [23].

Although most patients in the ICU have a central venous catheter in place, the peripheral venous cannula (PVC) is also a means of providing vascular access through which transfusion to critically ill patients can be administered. Based on several criteria, nurses independently decide on the size of the PVC which will be placed in the patient. The recommendation for the application of routine transfusion of most blood products is that the size of PVC should be from 20 to 24 gauge (G), while for rapid transfusion, the recommendation is that the PVC should be of a larger diameter, size 18 to 20 G [34]. For routine use of RBCs, a PVC size of 20 to 22 G is recommended due to the reduced risk of haemolysis, and for rapid transfusion, RBC size should be from 16 to 18 G [15].

More than two-thirds of our ICU nurses correctly answered the item about the size of the PVC in routine transfusion. The answers of other nurses were consistent with those of nurses in studies where a few nurses gave the correct answer to this item [20,26]. Namely, in our study, as in studies of Jogi et al. and Uzun, further analysis determined that nurses opted for cannulas of a significantly larger diameter, even in a routine, not an emergency, transfusion. However, although a PVC with a larger diameter will enable faster transfusion flow, at the same time, it will significantly increase the risk of mechanical phlebitis [34].

The results of our study indicate that ICU nurses and nurses from other clinical departments generally have a high level of knowledge about solutions compatible with blood products and how to administer drugs during transfusion. This finding is similar to previously published research [17,19,23,24,26].

Most standard administrative transfusion sets have integrated filters from 170 to 260 microns, with a maximum capacity of 4 units of RBCs [34]. Most care systems and transfusion administration sets are replaced following the manufacturer’s recommendations. In order to prevent the proliferation of microorganisms, the general recommendation is to change the blood administration set for continuous transfusion every 12 h [15,34]. The nurses’ knowledge in our study was deficient about blood administration set replacement when administering 2 to 4 units of RBCs and during continuous transfusion. Namely, most nurses who did not give the correct answer (from 2 to 4 units of RBCs and 12 h in case of continuous transfusion) indicated that regardless of the number of units the patient receives, a new blood administration set is used for each unit of blood product. From a financial perspective, this practice is questionable because it additionally increases the costs of the patient’s stay in the ICU. At the same time, it is also questionable from the aspect of the risk of HAI because with each replacement of the set, the number of manipulations with the luer extension of the vascular access device (PVC or central venous catheter) increases.

If signs of transfusion reactions do not appear during the first 15 min of transfusion administration, the flow rate of 50 mL/h is increased to meet the total administration time [15,34]. In our study, nurses had a low level of knowledge on items about blood transfusion flow rate. Similar results were obtained in previously published studies [17,18,20,22]. Also, more than half of the nurses in our study did not correctly answer the item about the maximum time required for RBC administration. In contrast, a significantly larger number of them (73.9%) answered that if the time for blood administration has expired (duration of transfusion longer than 4 h), the administration of blood should be stopped, and the anaesthesiologist–intensivist should decide whether to prescribe another unit.

It is well known that in the early stage of deterioration of the patient’s condition, as a response to inadequate oxygenation, the frequency of respiration and pulse increases, while the values of peripheral oxygen saturation (SpO2) and arterial blood pressure are without significant changes [35].

We find that the nurses’ level of knowledge regarding assessing vital signs at the beginning of transfusion administration (71.2%) and monitoring unconscious patients (72.1%) was satisfactory. However, they had a significantly lower level of knowledge about the observation time interval (57.7%) and the mandatory vital signs monitored during transfusion (48.6%). Since this was a multiple-choice item, we observed through detailed analysis that respiration rate was often not recognised as a vital function monitored during transfusion. According to the literature data, although it can contribute to the early recognition of dehydration in a patient, nurses often omit the assessment of breathing in clinical practice [36]. Such practice increases the risk that the patient’s deterioration is not recognised in time.

The lowest number of correct answers we received in this study were on the item about administering a transfusion to a febrile patient. This rate of correct responses may have been attributed to the fact that nurses do not consider decision-making about therapeutic procedures as their responsibility, nor collaborative, but solely the physician’s responsibility.

Adequate patient observation and monitoring of vital signs in all transfusion steps contribute to early detection and effective treatment of transfusion reactions [15]. Most of our nurses knew that urticarial rash usually occurs with a mild allergic reaction. However, regarding acute haemolytic reaction (AHTR), a significantly lower number of nurses answered correctly. Namely, 51 (45.9%) nurses correctly marked the symptoms and signs of AHTR. At the same time, every third nurse (32.4%) answered correctly about the nursing management of this serious and potentially life-threatening complication. The implication of the authors of studies where nurses had a knowledge deficit in the domain of the AHTR is that additional, periodically repetitive education is necessary [17,19]. A similar conclusion was made in the study of Uzun, where the results showed that the nursing management of transfusion reactions was well known to nurses [26]. Such a conclusion is not surprising because patient safety is a key aspect of all procedures during transfusion [15].

According to our results, nurses’ education level is a predictor of success on the transfusion knowledge test: nurses with a higher level of education had significantly better knowledge about transfusion compared to nurses with a lower level of education. This correlates with earlier conducted research [13,19,22,25]. Our findings showed that none of the ICU nurses with a higher level of education had a low level of knowledge about transfusion. Such results are not surprising from the perspective of the scope of the content of the programs’ curricula at different levels of education.

Encan and Dubey reported that nurses with more work experience had better knowledge about transfusion [19,22]. We obtained similar results. Working experience was a significant predictor of transfusion knowledge in our sample. Nurses with five or more years of work experience had the best knowledge, followed by those with less than one year and at least one to five years of work experience. In contrast, Panchawagh [32] and colleagues determined that nurses with one to five years of work experience had significantly better knowledge. In the same study, nurses with more than five years of experience had the lowest level of knowledge. The authors cite forgetting fundamental knowledge as one of the possible causes [32].

Inadequate knowledge is only one of the barriers to implementing safe transfusion practices. Hijji et al. [18] suggest that for the maximum application of this practice, it is necessary to overcome both personal obstacles by developing positive attitudes and many organisational obstacles, such as the number of nurses and material resources. Smith et al. [37] confirmed that carefully designed structured transfusion education programs have long-term positive effects. However, it was revealed that nurses’ knowledge levels did not decrease over time, but their attitudes about safe transfusion practice changed positively.

### Limitations

This study has strengths, such as providing significant data on ICU nurses’ transfusion knowledge, having a sufficient sample, and having a questionnaire with many items that panel experts revised, but it has certain weaknesses. First, the study was conducted in only one institution with a cross-sectional design, which limits the generalisation of the results. Also, although the study’s authors tried to include as many transfusion-related procedures of ICU nurses as possible in the questionnaire, some procedures may not be included.

## 5. Conclusions

The results of this study indicate that most Serbian ICU nurses had a moderate or low knowledge of transfusion. At the same time, a high deficit of knowledge was determined for the procedures of marking the blood sample tube, the rate of transfusion flow, the time of replacement of the transfusion administration set, and the procedures of observation and nursing management of transfusion reactions. Nurses with a higher level of education and longer working experience had significantly better knowledge. The results can be the basis for creating structured continuous education, which would increase knowledge, improve practice, and reduce risks to patient safety during the transfusion procedure.

## Figures and Tables

**Table 1:** Sociodemographic characteristics of the study sample (in total and by level of education).

	N (%)		
Variable	All Nurses	SMS	BA
Gender			
Male	18 (16.2)	17 (19.8)	1 (4.0)
Female	93 (83.8)	69 (80.2)	24 (96.0)
Age (years)			
19–28	53 (47.7)	50 (58.1)	3 (12.0)
29–38	36 (32.4)	18 (20.9)	18 (72.0)
39–48	19 (17.1)	15 (17.4)	4 (16.0)
≥49	3 (2.7)	3 (3.5)	0 (0.0)
Work experience			
<1	38 (34.2)	29 (33.7)	9 (36.0)
1–5	36 (32.4)	31 (36.0)	5 (20.0)
≥5	37 (33.3)	26 (30.2)	11 (44.0)

SMS = Secondary medical school; BA = Bachelor’s Degree.

**Table 2:** Distribution of nurses’ correct answers to items about knowledge related to blood and blood products transfusion procedures.

Items Based on Knowledge of Correct Responses n (%)	
I. Pretransfusion steps	
Verification of the medical order for transfusion and patient identification	80 (72.1)
2. Data, time, and place of labelling of blood sample test tubes for pretransfusion tests	26 (23.4)
3. The blood products transportation from the blood bank to the ICU	88 (79.3)
4. The transport of platelet concentrate from the blood bank to the ICU	65 (58.6)
5. Thawing of fresh frozen plasma (FFP)	78 (70.3)
6. Storage of red blood cells (RBCs) in a refrigerator	53 (47.7)
7. The time within unused blood units can be returned to the blood bank	60 (54.1)
8. Refreezing of once-thawed FFP	90 (81.1)
9. Blood product with the highest risk of contamination	41 (36.9)
II. Initiating the transfusion	
10. Verification of the accuracy of the transfusion product and the patient’s identity	110 (99.1)
11. Vital signs measured before administration of blood and blood products	79 (71.2)
12. A blood product that does not require a system with an integrated filter	90 (81.1)
13. Peripheral venous cannula (PVC) size for routine transfusion in ICU	80 (72.1)
14. Intravenous solution compatible with blood	99 (89.2)
15. Blood transfusion flow rate at the initiation of administration	59 (53.2)
16. The same transfusion administration set can be applied for the transfusion of 2 to 4 units of RBCs.	50 (45.0)
17. Usage of personal protective equipment—self-protection of the nurses from blood-borne infection	67 (60.4)
III. Administration of transfusion and transfusion reactions	
18. Flow rate of transfusion after the first 15 min from the start of administration	51 (45.9)
19. Monitoring of a sedated patient in the ICU during transfusion	109 (98.2)
20. Time interval of patient observation in the ICU during transfusion administration	64 (57.7)
21. Safe procedure for drug administration during transfusion of blood and blood products	98 (88.3)
22. Mandatory vital signs measured during transfusion of blood and blood products	54 (48.6)
23. The usage of blood transfusion in the ICU in a febrile patient	18 (16.2)
24. Maximum duration of using a transfusion administration set for continuous transfusion	38 (34.2)
25. The maximum time for transfusion of RBCs	48 (43.2)
26. The procedure with the blood and blood products when they were not applied within the stipulated time	82 (73.9)
27. Symptoms and signs of mild allergic transfusion reaction	101 (91.0)
28. Symptoms and signs of an acute haemolytic reaction (AHTR)	51 (45.9)
29. Initial nursing procedures in AHTR	36 (32.4)

**Table 3:** Score on the knowledge test—mean values of the total score and individual domains.

Knowledge TEST Score	Minimum	Maximum	Mean	SD
Total score (0–29)	10.00	27.00	17.70	4.28
Pretransfusion steps (0–9)	2.00	9.00	5.23	1.58
Initiating the transfusion (0–8)	3.00	8.00	5.71	1.32
Administration of transfusion and transfusion reactions (0–12)	3.00	11.00	6.29	1.96

SD = standard deviation.

**Table 4:** Distribution of nurses according to levels of knowledge about transfusion and sociodemographic characteristics.

	Knowledge Level		
	Low (0–15)	Moderate (16–22)	High (23–29)
	n (%)	n (%)	n (%)
Total	26 (23.4)	64 (57.7)	21 (18.9)
Gender			
Male	1 (3.8)	17 (26.6)	0 (0.0)
Female	25 (96.2)	47 (73.4)	64 (100)
Educational level			
SMS	26 (100.0)	59 (92.2)	1 (4.8)
BA	0 (0.0)	5 (7.8)	20 (95.2)
Work experience (in years)			
<1	16 (61.5)	16 (25.0)	6 (28.6)
1–5	10 (38.5)	22 (33.4)	4 (19.0)
≥5	0 (0.0)	26 (46.0)	11 (52.4)

SMS = Secondary medical school; BA = Bachelor’s Degree.

**Table 5:** Nurses’ knowledge test scores differences to nurses’ sociodemographic characteristics.

Sociodemographic Characteristic	Total Score				Pretransfusion Steps				Initiating the Transfusion				Administration of Transfusion and Transfusion Reactions			
Gender	M ± SD	U	*p*	r	M ± SD	U	*p*	r	M ± SD	U	*p*	r	M ± SD	U	*p*	r
Male	17.72 ± 1.93	835.00	0.987	ns	4.89 ± 1.13	707.00	0.288	ns	6.16 ± 1.04	654.00	0.132	ns	6.22 ± 1.11	797.00	0.746	ns
Female	17.69 ± 4.61				5.30 ± 1.65				5.62 ± 1.35				6.31 ± 2.10			
Educational level	M ± SD	t (df)	*p*	d	M ± SD	t (df)	*p*	d	M ± SD	t (df)	*p*	d	M ± SD	t (df)	*p*	d
SMS	16.03 ± 3.15	11.022 (109)	0.000	0.53 *	4.62 ± 1.07	11.053 (109)	0.000	0.53 *	5.43 ± 1.32	4.504 (109)	0.000	0.16 *	5.65 ± 1.57	8.086 (109)	0.000	0.38 *
BA	23.44 ± 2.14				7.36 ± 1.15				6.68 ± 0.75				8.51 ± 1.53			
Work experience (in years)	M ± SD	F (df)	*p*	η^2^	M ± SD	F (df)	*p*	η^2^	M ± SD	F (df)	*p*	η^2^	M ± SD	F (df)	*p*	η^2^
<1	16.63 ± 5.17	9.201 (110)	0.000	0.15 *	5.11 ± 1.99	0.863 (110)	0.425	ns	5.31 ± 1.56	14.467 (110)	0.000	0.21 *	5.82 ± 1.82	8.636 (110)	0.000	0.14 *
1–5	16.47 ± 3.65				5.08 ± 1.27				5.25 ± 0.94				5.75 ± 1.76			
≥5	20.00 ± 2.71				5.51 ± 1.37				6.56 ± 0.93				7.32 ± 1.93			

M = mean; SD = standard deviation; U = Mann–Whitney test; *p*-value; r-value; ns = not significant; t = *t*-test; df = degrees of freedom; d = Cohen’s d indicator (* large effect); F = ANOVA; η^2^ = partial eta squared; SMS = Secondary medical school; BA = Bachelor’s Degree.

**Table 6:** Standard multiple regression model for the prediction of the total score.

	Unstandardised Coefficient		Standardised Coefficient	t-Value	*p*-Value	Correlations Part		
	ß	SE	Beta				F	*p*-Value
Constant	5.926	0.986		6.010	0.000			0.000
Level Education	7.237	0.617	0.710	11.731	0.000	0.708	83.152	
Work experience	1.461	0.314	0..282	4.661	0.000	0.281		

ß = coefficient; SE= Standard Error; F = ANOVA.

## Data Availability

All data relevant to the study are included in the article.
